# Effectiveness of the Eggs Make Kids demand‐creation campaign at improving household availability of eggs and egg consumption by young children in Nigeria: A quasi‐experimental study

**DOI:** 10.1111/mcn.13447

**Published:** 2022-11-08

**Authors:** Leila M. Larson, Edward A. Frongillo, Bezawit E. Kase, Lynnette M. Neufeld, Wendy Gonzalez, Irowa Erhabor, Eric W. Djimeu

**Affiliations:** ^1^ Department of Health Promotion, Education, and Behaviour University of South Carolina Columbia South Carolina USA; ^2^ Department of Epidemiology and Biostatistics University of South Carolina Columbia South Carolina USA; ^3^ Food and Agriculture Organization of the United Nations (FAO) Viale delle Termi de Caracalla Rome Italy; ^4^ Global Alliance for Improved Nutrition Geneva Switzerland; ^5^ Global Alliance for Improved Nutrition Abuja Jabi Nigeria; ^6^ Global Alliance for Improved Nutrition Washington District of Columbia USA

**Keywords:** child nutrition, demand‐creation campaign, egg, Nigeria

## Abstract

Using a quasi‐experimental design, our study aimed to determine the effectiveness of the ‘Eggs Make Kids Sharp & Bright and Strong & Active’ demand‐creation campaign in Nigeria. The intervention arm received emotionally compelling radio and television advertisements about eggs, and was exposed to promotional activities and advertising about eggs at points of purchase, schools and health facilities; the comparison arm received no intervention. Children 6–59 months of age (intervention: *n* = 1359; comparison: *n* = 1485) were assessed 14 months apart. Intent‐to‐treat analyses with analysis of covariance method assessed the impact of the intervention on caregivers' behaviour towards eggs, caregivers' willingness to pay for eggs, availability of eggs in households, and consumption of eggs by children 6–59 months of age. Analyses were adjusted for possible confounders and perceived effects of COVID‐19 on finances and food consumption. Compared to the comparison arm, the intervention arm showed a greater prevalence of household egg acquisition (odds ratio = 1.34, *p* < 0.0001), and larger improvements in caregiver self‐efficacy (*β* = 0.242, *p* = 0.004) and intent to feed eggs to children (*β* = 0.080, *p* = 0.021). No effects were found on children's egg consumption or caregivers' reported willingness to pay for eggs. The lack of impact on child egg consumption despite increased acquisition of eggs and caregiver self‐efficacy suggests that other barriers to child consumption may exist. Additional research should further investigate factors that may influence intrahousehold distribution of eggs and whether these may also influence other nutritious foods.

## Key messages

- An emotionally engaged demand creation campaign around eggs resulted in small improvements in household acquisition of eggs and caregivers' self‐efficacy and intent to feed eggs to their child 6–59 months of age, but did not improve egg consumption in children.
- Economic hardships imposed by the COVID‐19 pandemic and low interpersonal communication associated with the campaign may have contributed to the lack of effects of egg consumption.
- Demand generation activities as stand‐alone activities are insufficient in improving egg consumption among children 6–59 months of age in this context.

## Graphical abstract

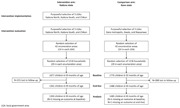

## 1 INTRODUCTION

Given the important role that nutrition in early life plays in children's health and development, Nigeria's high rates of childhood undernutrition constitute an important public health problem. According to the 2018 Nigeria Demographic and Health Survey, 37% of children 6–59 months of age are stunted and 68% are anaemic. Furthermore, only 11% of children 6–23 months of age are fed minimally acceptable diets (NPC & ICF, 2019).

The consumption of nutrient‐dense foods, such as eggs, can improve children's growth and development (Garg et al., 2020). Eggs provide essential fatty acids, proteins, choline, vitamins and minerals necessary for the developing child (Iannotti et al., 2014). In a randomized controlled trial in Ecuador, children 6–9 months of age who were provided with one egg per day for 6 months showed improved linear growth compared with their peers in the control arm (Iannotti et al., 2017). A study in Malawi that provided one egg per day to children 6–15 months of age for a duration of 12 months found improvements in child development domains among children who were less vulnerable at baseline (Prado et al., 2020). In Nigeria, however, only 13% of breastfed and 28% of nonbreastfed children are fed eggs (NPC & ICF, 2019).

Nutrition education interventions have been shown to increase consumption of eggs. For instance, nutrition education in rural China encouraging the consumption of eggs during the complementary feeding period was associated with increased consumption of eggs by children 4–12 months of age (Guldan et al., 2000). At the time of the study, the World Health Organization recommended initiating complementary foods at 4–6 months of age, in 2001 the recommendation was revised to 6 months of age (WHO, 2001). In a single‐arm study in Indonesia, a social marketing campaign promoting dark green leafy vegetables and eggs was associated with an increase in egg consumption in children 12–36 months of age and their mothers (de Pee et al., 1998).

While nutrition education interventions facilitate learning experiences to influence nutrition‐related behaviours, demand creation programmes aim to create desire for certain foods among consumers and influence their choices at points of purchase. Little is known about the effects of demand‐creation campaigns around eggs, particularly in low‐ and middle‐income settings. The ‘Eggs Make Kids’ campaign was launched by the Global Alliance for Improved Nutrition (GAIN) in Nigeria in October 2019. Using commercial marketing techniques and an understanding of consumer behaviour, the campaign aimed to create demand for eggs for children 6–59 months of age using emotionally engaged messages. Employing mass media and point‐of‐purchase demand‐creation activities, the campaign encouraged parents to feed their children eggs. The evaluation of this demand‐creation campaign aimed to determine the effectiveness of the egg demand‐creation campaign in improving four outcomes:

1. consumption of eggs by children 6–59 months of age in the previous 7 days;
2. the availability of eggs in households in the previous month;
3. caregivers' knowledge, attitudes, and beliefs towards eggs, and;
4. caregivers' willingness to pay for eggs.

## 2 METHODS

### 2.1 Intervention description

Between October 2019 and December 2020, GAIN implemented an egg demand‐creation campaign targeted to families of children 6–59 months of age living in Kaduna state. In Kaduna, 47% of children under 5 years of age were stunted, 12% were wasted, and 34% were underweight (NBS & UNICEF, 2017). Furthermore, 41% of households in Kaduna consumed fewer than five food groups (FAO, 2016). Only 15.6% of children 6–23 months of age met dietary diversity standards, and just 13.9% had a minimally acceptable diet (NPC & ICF, 2014). Affordability studies showed that about 70% of the population in Kaduna State could afford two eggs per child on a weekly basis and eggs were an acceptable food to feed to children (LaRose et al., 2020). The decision to implement the intervention in Kaduna state was taken together with the government and other local counterparts. Administratively, Nigeria is divided into states. Each state is subdivided into local government areas (LGAs), and each LGA is divided into localities and further subdivided into enumeration areas. Three LGAs in Kaduna were purposefully selected to receive the demand‐creation campaign activities. The selection of the three LGAs (Kaduna north, Kaduna south and Chikun) in Kaduna was made based on the concentration of egg vendors and an analysis of affordability.

Campaign activities included above‐the‐line (ATL) marketing, which focuses on mass media promotion and reaching a large audience, as well as below‐the‐line (BTL) marketing, which focuses on reaching a smaller targeted audience. State‐wide ATL activities included television spots, cooking shows, radio spots, jingles, dramas and tricycle branding. These ATL activities were designed to emotionally engage caregivers and increase their confidence in choosing eggs for their children. BTL activities included point‐of‐purchase materials (such as paper and plastic posters and bunting, and banners and parasols bade of PVC flex and vinyl); neighbourhood, compound, and open market shows; and promotion through household visits. BTL activities were designed to engage with caregivers on a personal level and prompt consumers to choose eggs at the point of purchase. Community‐based engagement activities aimed to increase consumers' knowledge about the benefits of eggs and provide advice on how to prepare them. The campaign used the theme ‘eggs make kids strong and active, sharp and bright’ and intended to encourage parents to feed children at least two eggs per week. Further details on the campaign activities are presented by LaRose et al. (2020).

Several months after the October 2019 launch of the campaign, the COVID‐19 pandemic began, resulting in egg shortages in local markets due to a lack of supply, poor credit access and limited distribution (LaRose et al., 2020). In response, intervention implementers reallocated eggs to markets in the study area experiencing shortages. A repurposed truck, the ‘Mobile Egg Truck’ was used to pick up eggs from farms and wholesalers and deliver them to community markets. Other adaptations to the campaign included an increase in frequency of campaign radio messaging, an early launch of the tricycle branding activity to increase exposure to campaign, and rescheduling of BTL activities planned for the first months of the pandemic.

### 2.2 Intervention evaluation

The campaign evaluation design was quasi‐experimental, with intervention and comparison arms. Outcomes were assessed in both arms 14 months apart, before and after implementation of the intervention. Kano state was purposefully chosen as the comparison arm because it was noncontiguous with Kaduna state, the intervention arm, and the distance between them was large enough to result in minimal contamination. In Kano, the three purposefully selected comparison LGAs (Kano metropolis, Gwale and Nassarawa) had characteristics similar to those of the LGAs selected in Kaduna.

Stratified multi‐stage sampling was used to draw a representative sample of children 6 months to 5 years of age for inclusion in the intervention evaluation. In the first stage, simple random sampling using a random number generator was used to select 14 enumeration areas within each intervention and comparison LGA. In the second stage, a household listing was used to randomly sample 29 households from each of the enumeration areas. Households were eligible to participate in the evaluation if they had at least one child 6–59 months of age living in the project area; vulnerable families (i.e., families headed by children, homeless families living on the street in the community, families with a head of household with a severe cognitive disability) were excluded. All children 6–59 months of age within the selected households were eligible to participate in the evaluation.

All children enroled in the evaluation at baseline were to be reassessed at end‐line. Participants were considered lost to follow‐up because of migration out of the study area, imprisonment and death.

### 2.3 Sample size estimation

This study intended to include a total sample size of 2500 households. The sample size was based on the percentage of breastfed children aged 6–23 months who consumed at least one egg in the last day, which was estimated to be 13% (NPC & ICF, 2019). The sample size calculation was based on a minimum detectable difference of 6.5 percentage points (pp) (13% to 19.5%) in children consuming eggs, alpha of 0.05, power of 0.90, an average cluster size of 24 and an intracluster correlation of 0.025. This yielded a sample size of 1008 households in 42 clusters per arm. The sample size was increased by 20% to account for refusals and attrition.

### 2.4 Data collection

Written informed consent was obtained from all caregivers participating in the survey. An independent data collection firm (Oxford Policy Management) administered all baseline and end‐line surveys using face‐to‐face interviews. Baseline data collection occurred in September 2019 and end‐line collection in November and December 2020. At both visits, questionnaires were administered to caregivers of children included in the evaluation.

### 2.5 Outcomes

For the first outcome, we assessed (1) the change in the proportion of children consuming eggs in the previous 7 days, (2) the change in the proportion of children consuming at least two eggs in the previous 7 days, and (3) the change in the average number of times eggs were consumed in the previous 7 days from baseline to end‐line in the two study arms. For the second outcome, we assessed (1) the change in the proportion of households purchasing eggs in the previous month, and (2) the change in the proportion of households acquiring eggs in the prior week from baseline to end‐line in the two study arms.

For the third outcome, we assessed caregivers' attitudes, the perceived benefits of feeding eggs to young children, social norms around eggs, and caregivers' self‐efficacy and intentions with regard to feeding eggs to children. At baseline, factor analysis, assuming one factor to obtain a factor score, was used to construct measures of caregiver's attitude, perceived benefits of eggs for a child, self‐efficacy in terms of feeding eggs, perceived norms towards feeding eggs, and intention to feed eggs to children based on their respective items. Factor analysis coefficients from baseline were used to calculate end‐line factor scores for each outcome separately, which allowed the scores at each timepoint to be comparable. Also, an overall behaviour factor score was calculated using baseline factor analysis coefficients from variables on caregivers' attitudes, perceived benefits, norms, self‐efficacy and intent to feed eggs to child. We compared the change in the mean score from baseline to end‐line between the two study arms for caregivers' attitudes, perceived benefits, norms, self‐efficacy and intention to feed eggs separately, as well as the overall behaviour factor score.

For the fourth outcome, we assessed the change in mean maximum price that caregivers would be willing to pay for eggs from baseline to end‐line between the two study arms. At baseline and end‐line, respondents who had purchased eggs in the previous 30 days were asked about their willingness to purchase eggs at an incrementally higher cost (i.e., +3, +5, +10 naira). If the respondent was not willing to pay a higher price, the price they paid for eggs on their last purchase was assigned as their willingness to pay for eggs. Otherwise, the amount of the increment they affirmed was added to the amount they spent on eggs on their last purchase. Those who had not purchased eggs in the previous 30 days were left censored.

### 2.6 Other measurements

For each participant, information was collected on household and child sociodemographics; food acquisition, purchase, and consumption; decision making; and child health and appetite. Household food insecurity was assessed using the Household Food Insecurity Access Scale (Coates et al., 2007). Food diversity was measured as the number of complementary food groups consumed excluding eggs following WHO/UNICEF guidance (WHO & UNCEF, 2021). Social desirability bias (i.e., the tendency for respondents to give socially desirable responses instead of giving responses that are reflective of their true feelings or behaviours) was assessed at end‐line using a validated scale (Rawat et al., 2017). The scale included five items with yes/no response, and a sum score was created by adding up the socially desirable answers (scores ranged from 0 to 5, with higher scores indicating more socially desirable responses). Scores were categorized as very low (score = 0), low (score = 1), medium (score = 2), high (score = 3), and very high (score = 4–5).

A wealth index was calculated using principal component analysis with components that indicate family assets, type of household, land and livestock ownership, source of drinking water, and availability/type of toilet facilities; weights from baseline were applied to the same components at end‐line. Given that the intervention implementation overlapped with the COVID‐19 pandemic, the study included a series of questions at end‐line on the effects of the pandemic on households' financial situation, access to food and consumption of food.

Respondents from both the intervention and comparison arms reported exposure to egg messaging at baseline, indicating the presence of egg promotion materials in the area before the start of the intervention. Self‐reported exposure to promotional messages could thus not be used to accurately or reliably assess exposure to the intervention's promotional activities. Instead, we reasoned that exposure to the intervention's promotional activities would be associated with household distance to those activities. Using latitude and longitude of each household and of the location of all BTL activities, we calculated the minimum distance between each household and any BTL activity location, using a straight line. Distance was categorized according to whether it was less than 2 kilometres (km) to any BTL activity because it was plausible that caregivers living within 2 km of an activity would have been exposed to that activity. We repeated this calculation for the distance to any of the three most frequent BTL activities (point‐of‐sale material deployment, market shows and neighbourhood shows).

### 2.7 Statistical analyses

Descriptive data were presented as count and percentage for categorical variables, mean ± standard deviation (SD) for normally distributed continuous variables, and median and quartiles (25th and 75th) for non‐normally distributed variables. Continuous outcomes were normally distributed. Because of the quasi‐experimental design of the study, we tested for differences in baseline characteristics between intervention and comparison arms.

Intent‐to‐treat analyses were used to assess whether the intervention resulted in improvements in all outcomes using two standard methods: (1) difference‐in‐differences models with individual as a fixed effect and (2) the analysis of covariance (ANCOVA) method. The difference‐in‐differences method assumes uniform dependency on baseline values; it also assumes that the only event that occurred differentially between arms during the baseline to end‐line period was the intervention. The ANCOVA method relaxes the first assumption and can account for differences between arms in dependency on baseline values and differences in other events (McKenzie, 2012). Given that the intervention occurred during the COVID‐19 pandemic, we used the ANCOVA method as the primary analysis, accounting for differences between arms in the impact of the pandemic on household finances and food consumption.

Using the difference‐in‐differences method, the models incorporated as fixed categorical variables individual identification number, visit, intervention and the visit‐by‐intervention interaction. Using the ANCOVA method, the end‐line value of the outcome was regressed on the baseline value of the outcome and intervention arm. The ANCOVA method used the full analytic sample for binary outcomes, as opposed to the difference‐in‐differences method, in which only participants who showed change from baseline to end‐line were included in the analysis. For both methods, the intervention effect was estimated as the difference between intervention and comparison arms in mean change from baseline to end‐line.

Both methods used all the longitudinal data—that is, individuals with both baseline and end‐line assessments. Continuous outcomes were analyzed using a linear model. Binary outcomes were analyzed using a logit model. Ordinal outcomes were analyzed using ordinal logistic regression. Accounting for clustering of LGAs was done using complex sample procedures, designating LGA as the primary sampling unit. Because the LGA was the unit at which the intervention was assigned and implemented, variation and degrees of freedom among LGAs were used to construct tests for the difference‐in‐differences and ANCOVA methods. A mixed‐effect tobit model was used to examine the effect of the intervention on willingness to pay for eggs, using LGA and participant as random effects. The intervention effect was obtained as the interaction between intervention arm and visit.

To adjust for possible confounding by baseline characteristics that differed meaningfully between arms or had *p* < 0.1, backwards elimination was used to add these variables to the ANCOVA models that also adjusted for the effect of the pandemic. We conducted an ancillary analysis to examine whether restricting the intervention sample to households within a 2 km distance of BTL activities produced differential intervention effects. Subgroup analyses were run to examine whether intervention effects on outcomes 1 and 2 differed between subgroups. We added a subgroup‐by‐visit‐by‐intervention interaction term to the main analyses. The following subgroups were examined: (1) child sex (female/male), (2) household religion, (3) baseline food insecurity status (any/none), (4) baseline wealth (below/above median) and (5) baseline behavioural determinants (i.e., caregivers' attitudes, perceived benefits, norms, self‐efficacy, intention to feed eggs and behaviour factor score) (favourable/unfavourable). We also examined whether effects differed for children whose age at enrolment was 6 to <24 or ≥24 months using stratified analyses.

Possible bias because of social desirability was examined. We examined whether end‐line mean or prevalence of primary and secondary outcomes differentially improved with higher social desirability scores by state.

For instances when participants missed the end‐line study visit, no data were imputed. Attrition between study arms was compared. Data were analyzed using SAS 9.4 (SAS Institute) and Stata 16 (StataCorp LP).

### 2.8 Ethical statement

This study was approved by the National Health Research Ethics Committee, the Kaduna State Health Research Ethics Committee, and the Kano State Health Research Ethics Committee, and registered with the U. S. National Institutes of Health as a clinical trial (www.ClinicalTrials.gov; NCT04937218).

## 3 RESULTS

**Figure 1:**
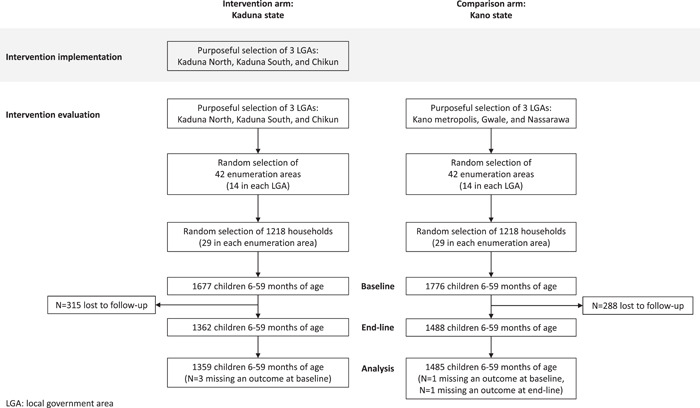
Study design

A total of 3453 children (from 2436 households) from LGAs in either the intervention or the comparison arm completed baseline assessments. Loss to follow‐up was 18.8% in the intervention arm and 16.3% in the comparison arm. An additional six children were missing primary outcome measures; therefore 2844 children were included in analyses (Figure 1). Baseline household and child characteristics did not differ between the intervention and comparison arms except for self‐reported exposure to messaging related to eggs (Table 1 and Supporting Information: Table 1). A total of 4.7% of caregivers in the intervention arm and 29.9% of caregivers in the comparison arm reported hearing information about eggs from the radio at baseline. Furthermore, at baseline 45.5% of caregivers in the intervention arm and 60.7% of caregivers in the comparison arm reported receiving the message that ‘Eggs make children strong/active or sharp/bright’. Exposure to information on eggs from clinics and other sources was low (<6%), but similar between arms.

**Table 1:** Baseline household and child characteristics

Characteristic	Intervention arm (*n* = 1359), *n* (%) or mean ± SD	Comparison arm (*n* = 1485), *n* (%) or mean ± SD	*p V*alue
Child and household demographics			
Child sex (male)	711 (52.3)	754 (50.8)	0.468
Child age (months)			0.285
6–23.9	413 (30.4)	423 (28.5)	
24–59.9	946 (69.6)	1062 (71.5)	
Religion			0.099
Christian	322 (23.7)	36 (2.4)	
Muslim	1037 (76.3)	1449 (97.6)	
Wealth index	−0.02 ± 0.97	0.02 ± 1.02	0.658
Household food insecurity (0–27)	7.6 ± 6.9	6.1 ± 6.9	0.149
Eggs available in markets (yes)	1235 (90.9)	1265 (85.2)	0.022
Eggs produced in household (yes)	163 (11.9)	169 (11.4)	0.749
Hens and layers owned (yes)	225 (16.6)	226 (15.2)	0.552
Pullets and chicks owned (yes)	79 (5.8)	100 (6.7)	0.629
Child feeding			
Food diversity (0–6, not including eggs)	3.1 ± 1.3	3.1 ± 1.4	0.847
Frequency of feeding child (daily)	3.4 ± 1.4	3.1 ± 1.3	0.001
Child's age at egg introduction (months)	8.3 ± 4.9	7.5 ± 3.8	0.013
Self‐reported exposure to messaging around eggs			
Caregiver heard information on eggs from the radio (yes)	64 (4.7)	445 (29.9)	0.003
Caregiver received information on eggs from other sources (yes)	49 (3.6)	83 (5.6)	0.243
Caregiver received information on eggs from clinics (yes)	42 (3.1)	36 (2.4)	0.436
Received information: eggs make children strong/active or sharp/bright (yes)	619 (45.5)	901 (60.7)	0.004

*Note*: Additional baseline characteristics are shown in Supporting Information: Table 1. Eggs were excluded from the food diversity score calculation because egg consumption was the study's primary outcome, and as such, was instead utilized in the intent‐to‐treat analyses presented in Table 3.

Abbreviation: SD, standard deviation.

At baseline, the mean (SD) reported price of eggs (in naira) was 33.45 (9.58) in the intervention state and 31.83 (10.70) in the comparison state. At end‐line, the mean reported price had increased to 42.65 (7.99) in the intervention state and 41.73 (8.50) in the comparison state.

### 3.1 Self‐reported exposure to intervention in Kaduna at end‐line

Caregivers were asked whether they had heard, seen, or participated in any of the campaign activities. At end‐line, self‐reported exposure was different between intervention and comparison arms (Table 2). Self‐reported exposure to any ATL activities was 32.0% in the intervention arm and 16.5% in the comparison arm; exposure to any BTL activities was 29.7% in the intervention arm and 8.3% in the comparison arm. Exposure to any ATL or BTL activities, and exposure to at least one ATL and one BTL activity were differential between arms.

**Table 2:** Self‐reported exposure to above‐the‐line (ATL) and below‐the‐line (BTL) activities at end‐line, by intervention group

Exposure	Intervention arm (*n* = 1359), *n* (%)	Comparison arm (*n* = 1485), *n* (%)
Exposed to any ATL activities	435 (32.0)	245 (16.5)
Exposed to any BTL activities	404 (29.7)	124 (8.3)
Exposed to any ATL or BTL activities	609 (44.8)	305 (20.5)
Exposed to at least one ATL and one BTL activity	230 (16.9)	64 (4.3)
Above‐the‐line activities		
Heard or seen any information or advertisement about eggs	380 (27.9)	299 (13.4)
Source: radio	142 (10.4)	131 (8.8)
Source: television	117 (8.6)	37 (2.5)
Heard or seen the following messages		
Eggs make kids strong and active	296 (2.2)	133 (9.0)
Eggs make kids sharp and bright	257 (18.9)	111 (7.5)
Eggs make kids' meal more delicious	123 (9.1)	43 (2.9)
Buy eggs for your children today	125 (9.2)	27 (1.8)
Give eggs to your children at least twice a week	112 (8.2)	55 (3.7)
Eggs help children have a better future	71 (5.2)	27 (1.8)
Eggs give energy to play soccer and run	50 (3.7)	9 (0.6)
Eggs help children have sharp and bright minds	87 (6.4)	34 (2.3)
Eggs are good for children	180 (13.2)	92 (6.2)
Seen Eggs Make Kids campaign logos	166 (12.2)	52 (3.5)
Heard Eggs Make Kids campaign song about eggs	167 (12.4)	80 (5.4)
Below‐the‐line activities		
Seen in the market		
Posters or banners with the message ‘Eggs make kids strong and active’ or ‘Eggs make kids sharp and bright’	291 (21.4)	75 (5.0)
Buntings or danglers with the message ‘Eggs make kids strong and active’ or ‘Eggs make kids sharp and bright’	47 (3.5)	11 (0.7)
Aprons, shirts, or caps with the message ‘Eggs make kids strong and active’ or ‘Eggs make kids sharp and bright’	78 (5.7)	17 (1.1)
Umbrellas with the message ‘Eggs make kids strong and active’ or ‘Eggs make kids sharp and bright’	65 (4.8)	23 (1.6)
Face masks with the message ‘Eggs make kids strong and active’ or ‘Eggs make kids sharp and bright’	11 (0.8)	0 (0)
Other	51 (3.8)	5 (0.3)
Participation in activities about eggs	19 (1.4)	1 (0.07)
Type: compound or neighbourhood show with music and egg recipe demonstration	4 (0.3)	0
Type: market show with music and talks about eggs	0	0
Type: household visit promoting eggs	2 (0.2)	1 (0.07)
Type: World Egg Day celebration	0	0
Receipt of materials about eggs		
Egg campaign flyers or pamphlets	2 (0.2)	3 (0.2)
Egg campaign gifts such as cooking spoons and bowls	2 (0.2)	0
Cooked or raw eggs as campaign gifts	2 (0.2)	0
Face masks or face shields	0	0
Hand sanitizers	0	0
T‐shirts	1 (0.1)	0
Colouring book, mug or pen	2 (0.2)	1 (0.07)
Other	1 (0.1)	2 (0.1)

### 3.2 Impact of COVID‐19

The reported impacts of the COVID‐19 pandemic on households' financial situation, food prices and egg prices did not differ between arms at end‐line, but the reported change in household consumption of food pre‐ versus post‐pandemic lockdown differed by arm. Self‐reported household‐level food consumption decreased in 58.9% of households in Kaduna and 39.2% of households in Kano; food consumption stayed consistent in 25.4% of households in Kaduna and 36.6% of households in Kano; and food consumption increased in 15.8% of households in Kaduna and 24.2% in Kano (Supporting Information: Table 2).

### 3.3 Intervention effects

Using the ANCOVA model adjusted for the differential effect of COVID‐19 between the two intervention arms, the mean change from baseline to end‐line in the number of times eggs were consumed by children in the previous 7 days was negative in both arms, but less so in the intervention than in the comparison arm (−0.13 in intervention arm compared with −0.18 in comparison arm) (Table 3 and Supporting Information: Table 3). Similarly, the change in the prevalence of children consuming eggs in the previous 7 days was more favourable in the intervention arm than in the comparison arm. These differences were not seen in the unadjusted ANCOVA analyses or the difference‐in‐differences analyses. When adjustment was made for covariates that were different between arms at baseline, changes in the number of times eggs were consumed and the prevalence of egg consumption were no longer different between arms (Table 3). No differences between intervention arms were observed for the prevalence of children consuming ≥2 eggs in the previous 7 days.

**Table 3:** Intervention effects using intent‐to‐treat sample and analysis of covariance method

Outcome	Intervention arm (*n* = 1359), mean (±SD) or *n* (%) at baseline	Comparison arm (*n* = 1485), mean (±SD) or *n* (%) baseline	Intervention arm (*n*= 1359), mean (±SD) or *n* (%) at end‐line	Comparison arm (*n*= 1485), mean (±SD) or *n* (%) at end‐line	Unadjusted regression coefficient or odds ratio (*p* Value)	COVID‐19‐adjusted^a^ regression coefficient or odds ratio (*p* Value)	Fully adjusted^b^ regression coefficient or odds ratio (*p* Value)
Number of times eggs were consumed in the previous 7 days	1.34 (±1.74)	1.31 (±1.75)	1.21 (±1.66)	1.13 (±1.57)	0.071 (0.332)	0.175 (0.044)	0.087 (0.318)
Children consuming at least one egg in the previous 7 days (yes)	715 (52.6%)	746 (50.2%)	649 (47.8%)	710 (47.8%)	0.969 (0.285)	1.14 (0.012)	0.967 (0.589)
Children consuming at least two eggs in the previous 7 days (yes)	496 (36.5%)	521 (35.1%)	462 (34.0%)	456 (30.7%)	1.16 (0.336)	1.37 (0.084)	1.23 (0.131)
Eggs purchased in the previous month (yes)	936 (68.9%)	992 (66.8%)	868 (63.9%)	968 (65.2%)	0.920 (0.372)	1.08 (0.385)	1.07 (0.384)
Eggs acquired in the previous 7 days (yes)	685 (50.4%)	731 (49.2%)	641 (47.2%)	619 (41.7%)	1.25 (0.0001)	1.45 (<0.0001)	1.34 (<0.0001)
Caregivers' behaviour factor score^c^	0.08 (±0.95)	−0.07 (±1.04)	0.44 (±0.88)	0.27 (±0.92)	0.157 (0.213)	0.198 (0.148)	0.096 (0.272)
Caregivers' attitude towards eggs	0.10 (±0.94)	−0.09 (±1.04)	0.59 (±0.80)	0.42 (±0.92)	0.160 (0.318)	0.186 (0.279)	0.174 (0.296)
Caregivers' perceived benefit of eggs	0.05 (±0.98)	−0.05 (±1.01)	0.31 (±0.85)	0.31 (±0.81)	0.0006 (0.995)	0.017 (0.885)	−0.048 (0.573)
Social norms	0.04 (±0.99)	−0.03 (±0.99)	0.29 (±0.83)	0.27 (±0.85)	0.022 (0.818)	0.044 (0.659)	0.038 (0.701)
Caregivers' self‐efficacy	0.05 (±1.05)	−0.05 (±0.94)	0.22 (±1.09)	−0.08 (±1.12)	0.289 (0.021)	0.325 (0.011)	0.242 (0.004)
Caregivers' intent to feed eggs	3.90 (±0.91)	3.81 (±0.91)	4.00 (±0.87)	3.88 (±0.91)	0.110 (0.073)	0.155 (0.027)	0.080 (0.021)

*Note*: Complex survey procedures were used to account for clustering at the local government area (LGA) level.

^a^ Adjusted for reported change in household consumption of foods due to COVID‐19 pandemic.

^b^ Adjusted for reported change in household consumption of foods due to COVID‐19 pandemic and for variables that were different between arms at baseline and remained in the multiple variable model with *p* < 0.1 following backward elimination.

^c^ The behaviour factor score was constructed from caregivers' attitudes towards feeding eggs to children, perceived benefits of eggs, self‐efficacy, social norms and intention to feeds eggs using factor analysis assuming one factor to obtain a factor score.

The change in the prevalence of households acquiring eggs in the previous 7 days from baseline to end‐line favoured the intervention arm (dropped by 3.2 pp in the intervention arm vs 7.5 pp in the comparison arm, *p* < 0.001). The change in the prevalence of households purchasing eggs in the previous 30 days from baseline to end‐line was not different between the intervention and comparison arms (dropped by 5.0 pp in the intervention arm vs 1.6 pp in the comparison arm, *p* = 0.384). Results did not differ across the ANCOVA and difference‐in‐differences analyses, unadjusted and adjusted for perceived effects of COVID‐19 and possible confounders (Table 3 and Supporting Information: Table 3).

The intervention resulted in improved caregiver self‐efficacy in terms of feeding eggs to a child and intention to feed eggs, using the ANCOVA analysis adjusted for COVID‐19. After further adjustment for covariates, only self‐efficacy remained different between intervention arms. The intervention had no effects on factor scores for caregiver attitudes towards eggs, perceived benefits of eggs for children, norms around eggs, or the overall behavioural factor score of these attitudes, knowledge, and beliefs pertaining to eggs (Table 3 and  Supporting Information: Table 3). No effects were found on respondents' willingness to pay for eggs (difference‐in‐differences estimate: −2.03, 95% CI: −5.27, 1.21)

### 3.4 Ancillary analyses accounting for exposure to intervention activities

Increasing distance to BTL activities (in km) was associated with lower odds of self‐reported exposure (OR = 0.944, *p* = 0.026). Restricting the intervention sample to households within 2 km of any BTL activity or to any of the three most frequent BTL activities resulted in slightly higher effects than the intent‐to‐treat analyses on all outcomes except caregiver self‐efficacy (Supporting Information: Table 4).

### 3.5 Effect modification analyses

The effect of the intervention on child consumption of at least one egg was modified by religion (*p* interaction = 0.042), and effects were larger in children in Muslim households than in Christian households (Muslim, OR = 0.973, *p* = 0.732); Christian, OR = 0.630, *p* = 0.011). Similarly, the effect of the intervention on child consumption of at least two eggs was modified by religion (*p* interaction < 0.001), showing that Muslim households (OR = 1.25, *p* = 0.110) benefited more from the intervention than Christian households (OR = 0.732 (*p* = 0.018). The effect of the intervention on egg acquisition was larger in households with a higher baseline intent to feed eggs (*p* interaction = 0.002). There was no differential effect based on the child's sex, baseline food insecurity, baseline wealth, and baseline attitudes, perceived benefits, norms and self‐efficacy.

A stratified analysis for children aged 6–24 months and for children aged 24 months and above at enrolment showed that the effect of the intervention did not differ between the two groups.

### 3.6 Social desirability bias

At end‐line, the mean (SD) for continuous outcomes and prevalence for categorical outcomes was not associated with categories of social desirability between arms (Supporting Information: Table 5).

## 4 DISCUSSION

This study implemented an emotionally engaged, large‐scale, 14‐month campaign to create demand for eggs, a nutritious food, in Nigeria. An evaluation of the intervention demonstrated improvements in household acquisition of eggs and caregivers' self‐efficacy and intent to feed eggs to their child 6–59 months of age. The impact was smaller than hypothesized, however, with no improvements in egg consumption, household purchasing of eggs, caregivers' attitudes towards eggs, perceived benefits of eggs, norms around eggs or willingness to pay for eggs.

Self‐efficacy, or a caregiver's judgement regarding their capability to utilize skills to feed eggs to their children, is an important step in changing future behaviour (RGN & RGN, 2002). Improved self‐efficacy for feeding eggs to children and increased egg acquisition, however, were not sufficient to change egg consumption in children. This may be due to economic hardships faced by households during the pandemic. For instance, qualitative research reinforced that egg prices were a deterrent to egg purchasing and consumption. The intervention monitoring data found an 18% increase in egg prices during the campaign's implementation period, with vendors reporting a 22%–68% decrease in egg sales between October 2020 and October 2019. Furthermore, we understand from trials in Ecuador and Malawi that providing eggs to households free of charge increases egg consumption among children with minimal compliance issues (Iannotti et al., 2017; Stewart et al., 2019).

The smaller than anticipated effects of the intervention could also be due to limitations in the design of the intervention, which saw low exposure to BTL activities, such as posters and banners, neighbourhood shows and household visits promoting eggs. Interventions such as this one may benefit from coupling their ATL and BTL activities with interpersonal communication from, for example, trusted sources of information such as community health workers and religious leaders, to encourage changes in intrahousehold allocation of eggs and the transition from caregiver awareness and self‐efficacy to the practice of feeding eggs to children. In a recent study in Nigeria, complementary feeding social and behaviour change communication targeting both fathers and mothers increased children's consumption of eggs, fish and minimum meal frequency (Flax et al., 2022). This type of intervention illustrates the importance of directing messages at fathers and of interpersonal communication through community meetings, religious services and home visits, in addition to mass media, to effectively influence behaviour change. Furthermore, our study's intervention was unable to overcome social norms associated with who in the household is prioritized for egg consumption. For instance, qualitative interview findings revealed that better‐quality foods were sometimes reserved for the men in the household (Blum, 2021). Future campaigns may benefit from conducting formative research on key cultural and context‐specific beliefs and norms around eggs so that campaigns and other strategies can be designed to effectively address the norms that act as barriers to children's egg consumption (Robert et al., 2021). In Ecuador, formative research helped inform the design of an effective social marketing campaign to promote eggs (Gallegos‐Riofrío et al., 2018). Similarly, other studies in India (Bentley et al., 2014) and Burkina Faso (Nordhagen & Klemm, 2018) were able to overcome negative cultural beliefs around egg consumption in children.

Collateral factors, such as high prices of eggs and additional increases in prices due to the COVID‐19 pandemic, could have also influenced the results. Qualitative interviews with mothers of children in the intervention areas showed that mothers were generally enthusiastic about the campaign and that the messages were acceptable (Blum, 2021). Lack of money was reported as the main barrier to increasing egg consumption, however, and some mothers expressed resistance to feeding eggs to their children because they did not want their children to become accustomed to eating eggs regularly when the household could not afford it. Furthermore, in the comparison and intervention arms, the average price of eggs increased by 31% and 27%, respectively. In the comparison arm, household acquisition of eggs decreased by 15%, representing an elasticity of −0.49. In contrast, in the intervention arm, household acquisition of eggs decreased by only 6%, representing an elasticity of −0.23. That is, the egg promotion campaign dampened the effect of increased egg prices on household acquisition of eggs.

In July 2019, Alive & Thrive launched a campaign in Kaduna state promoting complementary feeding, including egg consumption among children (Flax et al., 2022), which could explain the high rates of self‐reported exposure to egg promotion messaging in both states at baseline, as the two states are geographically close and the mass media campaign in Kaduna (i.e., television and radio spots) may have reached our study's comparison state of Kano. Similar spillover of mass media messages directed at the intervention state reaching the comparison state could have occurred in our study. Therefore, we ran an ancillary analysis restricted to those living within 2 km of the BTL promotional activities, such as point‐of‐sale materials, market shows, and neighbourhood shows, as an objective proxy for exposure. This method was biased neither by egg promotion activities in the study area unrelated to the intervention nor by caregiver recall. Results indicated slightly larger effects from the intervention among those who lived closer to the promotional activities, which implies that more frequent exposure to messages about eggs resulted in more beneficial behaviour and attitude change towards eggs.

## 5 CONCLUSION

The intervention was designed with the knowledge that prevalence of child malnutrition was high and diet quality poor (NPC & ICF, 2014) and egg consumption can improve child malnutrition (Garg et al., 2020; Iannotti et al., 2014, 2017). It hypothesized that the campaign would improve caregivers' motivation and intent to feed eggs to children in their household among those who could afford to purchase eggs. Despite the challenges imposed by the COVID‐19 pandemic on intervention delivery and the resulting limited exposure to BTL promotional activities, we conclude that demand generation activities as stand‐alone activities are insufficient in improving egg consumption among children 6–59 months of age in this context. Enhanced intensity and reach of the campaign, coupling of ATL and BTL activities with interpersonal communication, and complementary strategies to remove economic barriers to egg consumption may lead to larger and sustained behaviour change and norms around child egg consumption.

## AUTHOR CONTRIBUTIONS

Leila M. Larson, Wendy Gonzalez, Irowa Erhabor and Eric W. Djimeu designed the study. Leila M. Larson and Edward A. Frongillo designed the statistical analyses; Bezawit E Kase analyzed the data; Leila M. Larson drafted the manuscript; all authors critically reviewed and edited the manuscript.

## CONFLICT OF INTEREST

The authors declare no conflict of interest.

## Supporting information

Supplementary information.

## Data Availability

Data available on request from the authors.
