# Supplementary material for: Effectiveness of the Eggs Make Kids demand‐creation campaign at improving household availability of eggs and egg consumption by young children in Nigeria: A quasi‐experimental study
Source: Matern Child Nutr. 2022 Nov 8;19(1):e13447. doi: 10.1111/mcn.13447 (PMC9749590; doi:10.1111/mcn.13447)
Supplement: Supplementary file 1 — Supplementary information. [file MCN-19-e13447-s001.docx]

Supplemental Table 1: Baseline household and child characteristics

| **Characteristic** | **Intervention arm (n = 1,359),**  **n (%) or mean ± SD** | **Comparison arm (n = 1,485),**  **n (%) or mean ± SD** | ***p*-value** |
| --- | --- | --- | --- |
| Child sex (male) | 711 (52.3) | 754 (50.8) | 0.468 |
| Child age (months) |  |  | 0.285 |
| 6–23.9 | 413 (30.4) | 423 (28.5) |  |
| 24–59.9 | 946 (69.6) | 1062 (71.5) |  |
| Head of household (male) | 1,342 (99.1) | 1,471 (99.3) | 0.520 |
| Head of household age | 44.6 ±11.2 | 47.5 ±12.7 | 0.449 |
| Head of household education |  |  | 0.144 |
| No education | 118 (8.7) | 299 (20.7) |  |
| Primary | 161 (11.9) | 146 (9.9) |  |
| Secondary | 692 (51.1) | 562 (38.1) |  |
| Higher education | 383 (28.3) | 468 (31.7) |  |
| Spouse’s age | 32.7 ±8.7 | 33.9 ±9.2 | 0.071 |
| Spouse gender (male) | 17 (1.3) | 10 (0.7) | 0.343 |
| Spouse’s education |  |  | 0.153 |
| No education | 173 (12.7) | 370 (24.9) |  |
| Primary | 239 (17.6) | 260 (17.5) |  |
| Secondary | 729 (53.7) | 676 (45.6) |  |
| Higher education | 217 (15.9) | 176 (11.9) |  |
| Religion |  |  | 0.099 |
| Christian | 322 (23.7) | 36 (2.4) |  |
| Muslim | 1,037 (76.3) | 1,449 (97.6) |  |
| Wealth Index | -0.02 ±0.97 | 0.02 ±1.02 | 0.658 |
| Household Food Insecurity (0 to 27) | 7.6 ±6.9 | 6.1 ±6.9 | 0.149 |
| Awareness of government program providing eggs to students |  |  | 0.147 |
| Not aware | 586 (43.1) | 303 (20.4) |  |
| Aware but does not participate | 434 (32.0) | 446 (30.0) |  |
| Aware and does participate | 339 (24.9) | 736 (49.6) |  |
| Child enrolled in Home Grown School Food Assistance Programme (Yes) | 97 (7.1) | 316 (21.3) | 0.001 |
| Child enrolled in Infant and Young Child Food Assistance Programme (Yes) | 41 (3.0) | 102 (6.9) | 0.058 |
| Child enrolled in Action Against Hunger Food Assistance Programme (Yes) | 49 (3.6) | 72 (4.8) | 0.090 |
| Child enrolled in Community Management of Acute Malnutrition Food Assistance Programme (Yes) | 37 (2.7) | 55 (3.7) | 0.424 |
| Distance to markets (minutes) | 16.3 ±29.6 | 17.7 ±30.2 | 0.358 |
| Frequency of market visits |  |  | 0.167 |
| Daily | 472 (34.7) | 580 (39.0) |  |
| 2–6 times per week | 329 (24.2) | 335 (22.5) |  |
| Once per week | 237 (17.4) | 122 (8.2) |  |
| Once per 2 weeks | 128 (9.4) | 94 (6.3) |  |
| Once per month or less | 193 (14.2) | 354 (23.8) |  |
| Eggs available in markets (Yes) | 1,235 (90.9) | 1,265 (85.2) | 0.022 |
| Eggs produced in household (Yes) | 163 (11.9) | 169 (11.4) | 0.749 |
| Hens and layers owned (Yes) | 225 (16.6) | 226 (15.2) | 0.552 |
| Pullets and chicks owned (Yes) | 79 (5.8) | 100 (6.7) | 0.629 |
| Other poultry owned (Yes) | 61 (4.5) | 56 (3.8) | 0.579 |
| Child consumed egg in the previous 7 days (Yes) | 715 (52.6) | 747 (50.3) | 0.388 |
| Number of times child consumed eggs in the previous 7 days | 1.3 ±1.7 | 1.3 ±1.7 | 0.163 |
| Attitudes toward eggs | 0.09 ±0.93 | -0.09 ±1.04 | 0.012 |
| Perceived benefits of eggs | 0.05 ±0.98 | -0.05 ±1.01 | 0.246 |
| Caregiver’s self-efficacy | 0.05 ±1.05 | -0.05 ±0.94 | 0.279 |
| Social norm | 0.03 ±1.0 | -0.03 ±0.99 | 0.107 |
| Caregiver’s intent to feed eggs | 3.9 ±0.91 | 3.81 ±0.91 | 0.137 |
| Behavior factor score^a^ | 0.08 ±0.95 | -0.07 ±1.04 | 0.058 |
| Money caregiver willing to spend on eggs (Naira) | 30.5 ±20.9 | 29.1 ±22.5 | 0.073 |
| Caregiver heard information on eggs from the radio (Yes) | 64 (4.7) | 445 (29.9) | 0.003 |
| Caregiver received information on eggs from other sources (Yes) | 49 (3.6) | 83 (5.6) | 0.243 |
| Caregiver received information on eggs from clinics (Yes) | 42 (3.1) | 36 (2.4) | 0.436 |
| Received information: eggs make children strong/active or sharp/bright (Yes) | 619 (45.5) | 901 (60.7) | 0.004 |
| Decision maker on special child food purchases |  |  | 0.021 |
| Head of the household | 491 (36.0) | 775 (52.2) |  |
| Caregiver | 421 (31.1) | 298 (20.1) |  |
| Caregiver with others | 365 (26.9) | 274 (18.4) |  |
| Someone else | 82 (6.0) | 138 (9.3) |  |
| Decision maker on egg purchases |  |  | 0.017 |
| Head of the household | 534 (39.3) | 882 (59.4) |  |
| Caregiver | 422 (31.1) | 218 (14.7) |  |
| Caregiver with others | 329 (24.2) | 324 (21.8) |  |
| Someone else | 74 (5.4) | 61 (4.1) |  |
| Decision maker on feeding child |  |  | 0.794 |
| Head of the household | 366 (26.9) | 372 (25.1) |  |
| Caregiver | 589 (43.3) | 671 (45.2) |  |
| Caregiver with others | 358 (26.3) | 407 (27.4) |  |
| Someone else | 46 (3.4) | 35 (2.4) |  |
| Eggs purchased in the previous month (Yes) | 936 (68.9) | 992 (66.8) | 0.317 |
| Eggs acquired in the previous 7 days (Yes) | 685 (50.4) | 731 (49.2) | 0.609 |
| Chicken acquired in the previous 7 days (Yes) | 200 (14.7) | 253 (17.0) | 0.433 |
| Beef acquired in the previous 7 days (Yes) | 649 (47.8) | 607 (40.9) | 0.094 |
| Fish acquired in the previous 7 days (Yes) | 844 (62.1) | 671 (45.2) | 0.006 |
| Dairy acquired in the previous 7 days (Yes) | 1,011 (74.4) | 1,014 (68.3) | 0.208 |
| Food diversity (0 to 6, not including eggs) | 3.1 ±1.3 | 3.1 ±1.4 | 0.847 |
| Frequency of feeding child (daily) | 3.4 ±1.4 | 3.1 ±1.3 | 0.001 |
| Child’s age at egg introduction (months) | 8.3 ±4.9 | 7.5 ±3.8 | 0.013 |
| Child has immunization card |  |  | 0.024 |
| No | 120 (8.8) | 255 (17.2) |  |
| Yes | 1,225 (90.1) | 1,219 (82.1) |  |
| Don’t know | 14 (1.0) | 11 (0.74) |  |
| Child visited doctor in previous 6 months (Yes) | 237 (17.4) | 326 (21.9) | 0.087 |
| Child health and appetite |  |  | 0.922 |
| Both are not good | 20 (1.5) | 42 (2.8) |  |
| Either is not good | 64 (4.7) | 91 (6.1) |  |
| Both are very good | 1,275 (93.8) | 1,352 (91.0) |  |
| Fever in the previous 7 days (Yes) | 343 (25.2) | 633 (42.6) | 0.0002 |
| Diarrhea in the previous 7 days (Yes) | 178 (13.1) | 329 (22.1) | 0.039 |
| Cough or cold in the previous 7 days (Yes) | 503 (37.0) | 656 (44.2) | 0.038 |
| Shortness of breath in the previous 7 days (Yes) | 55 (4.1) | 149 (10.0) | 0.001 |

^a^ The behavior factor score was constructed from caregivers’ attitudes toward feeding eggs to children, perceived benefits of eggs, self-efficacy, social norms, and intention to feeds eggs using factor analysis assuming one factor to obtain a factor score.

Supplemental Table 2: Indicators of the impact of the COVID-19 pandemic, by intervention arm at end-line

| **Impact of COVID-19 pandemic indicators** | **Intervention arm**  **(n = 1,359),**  **n (%)** | **Control arm**  **(n = 1,485),**  **n (%)** |
| --- | --- | --- |
| Direction of change in food prices since COVID-19 pandemic |  |  |
| Going up | 1,343 (98.8) | 1,472 (99.1) |
| Staying about the same | 7 (0.5) | 6 (0.4) |
| Going down | 9 (0.7) | 7 (0.5) |
| Direction of change in egg prices since COVID-19 pandemic |  |  |
| Going up | 1,341 (98.7) | 1,459 (98.3) |
| Staying about the same | 13 (1.0) | 22 (1.5) |
| Going down | 5 (0.4) | 4 (0.3) |
| Household consumption of foods during the previous week compared with a typical week before state of emergency due to COVID-19 pandemic |  |  |
| More consumption of food now than before the lockdown | 214 (15.8) | 360 (24.2) |
| About the same amount of food consumption now as before the lockdown | 345 (25.4) | 544 (36.6) |
| Less consumption of food now than before the lockdown | 800 (58.9) | 581 (39.1) |
| Perceived household financial situation since COVID-19 pandemic |  |  |
| Worse than before COVID-19 | 1,248 (91.8) | 1,248 (84.0) |
| Pretty much the same | 81 (6.0) | 185 (12.5) |
| Better than before COVID-19 | 30 (2.2) | 52 (3.5) |

Supplemental Table 3: Intervention effects using intent-to-treat sample and difference-in-difference method^1^

| **Outcome** | **Intervention arm (n = 1,359),**  **mean change (± SD) or n (%) starting of those who changed** | **Comparison arm (n = 1,485),**  **mean change (± SD) or n (%) starting of those who changed** | **Difference between arms in changes from baseline to end-line**  **(coefficient or odds ratio [*p*-value])** | |
| --- | --- | --- | --- | --- |
|  |  |  | Unadjusted | Adjusted (for COVID-19)^a^ |
| Change in the average number of times eggs were consumed in the previous 7 days^b^ | -0.13 (±1.9) | -0.18 (±1.9) | 0.047 (0.584) | 0.094 (0.278) |
| Percentage of children starting to consume eggs in the previous 7 days (vs. stopping)^c,d^ | 209 (43.2%) | 250 (46.6%) | -3.4 (0.184) | -3.4 (0.781) |
| Percentage of children starting to consume ≥2 eggs in the previous 7 days (vs. stopping)^d,e^ | 189 (45.9%) | 188 (42.6%) | 3.3 (0.596) | 3.3 (0.305) |
| Percentage of households starting the purchase of eggs in the previous 30 days (vs. stopping)^d,f^ | 208 (42.9%) | 234 (47.6%) | -4.7 (0.334) | -4.7 (0.594) |
| Percentage of households starting the acquisition of eggs in the previous 7 days (vs. stopping)^d,g^ | 245 (45.9%) | 243 (40.6%) | 5.3 (0.028) | 5.3 (0.002) |
| Change in the average behavior factor score^h^ | 0.36 (±1.21) | 0.34 (±1.33) | 0.018 (0.904) | 0.025 (0.869) |
| Change in the average attitude score | 0.49 (±1.21) | 0.51 (±1.38) | -0.020 (0.875) | -0.016 (0.897) |
| Change in the average perceived benefit score | 0.26 (±1.26) | 0.35 (±1.29) | -0.09 (0.572) | -0.089 (0.590) |
| Change in the average norms score | 0.26 (±1.29) | 0.30 (±1.31) | -0.05 (0.683) | -0.048 (0.671) |
| Change in the average self-efficacy score | 0.17 (±1.44) | -0.04 (±1.39) | 0.204 (0.114) | 0.210 (0.099) |
| Change in the average intent to feed eggs score | 0.10 (±1.16) | 0.07 (±1.18) | 0.03 (0.647) | 0.046 (0.525) |

Note: Complex survey procedures were used to account for clustering at the local government area (LGA) level.

^a^ Adjusted for reported change in household consumption of foods due to COVID-19 pandemic (adjusted intent to treat).

^b^ 810 children in the intervention arm and 875 children in the comparison arm showed change from baseline to end-line in the number of times eggs were consumed.

^c^ 484 children in the intervention arm and 536 children in the comparison arm showed change from baseline to end-line in egg consumption (yes/no).

^d^ This outcome is a binary variable, and odds ratios with *p*-values are reported.

^e^ 412 children in the intervention arm and 441 children in the comparison arm showed change from baseline to end-line in consumption of at least 2 eggs (yes/no).

^f^ 484 households in the intervention arm and 492 households in the comparison arm showed change from baseline to end-line in egg purchase.

^g^ 534 households in the intervention arm and 598 households in the comparison arm showed change from baseline to end-line in egg acquisition.

^h^ The behavior factor score was constructed from caregiver’s attitudes toward feeding eggs to children, perceived benefits of eggs, self-efficacy, social norms, and intention to feeds eggs using factor analysis, assuming one factor to obtain a factor score.

COVID-19

Supplemental Table 4: Ancillary analyses restricting the intervention sample by proximity to location of below-the-line (BTL) activities

| **Outcome** | **Intent-to-treat analysis^a^**  (Exposed sample = 1,359) | **Distance from any BTL activity**^b^  (Exposed sample^c^ = 894) | **Distance from point-of-sale material deployment**  (Exposed sample^c^ = 656) | **Distance from neighborhood storm show**  (Exposed sample^c^ = 571) | **Distance from market storm show**  (Exposed sample^c^ = 472) |
| --- | --- | --- | --- | --- | --- |
| Number of times eggs were consumed in the previous 7 days^d^ | 0.175 (0.044) | 0.216 (0.021) | 0.226 (0.025) | 0.262 (0.008) | 0.326 (0.015) |
| Children consuming at least one egg in the previous 7 days (Yes)^e^ | 1.14 (0.012) | 1.14 (0.011) | 1.17 (0.048) | 1.16 (0.008) | 1.25 (0.044) |
| Children consuming at least two eggs in the previous 7 days (Yes)^e^ | 1.37 (0.084) | 1.43 (0.095) | 1.53 (0.054) | 1.57 (0.034) | 1.75 (0.005) |
| Eggs purchased in the previous month (Yes)^e^ | 1.08 (0.385) | 1.16 (0.115) | 1.12 (0.287) | 1.16 (0.167) | 1.19 (0.144) |
| Eggs acquired in the previous 7 days (Yes)^e^ | 1.45 (<0.0001) | 1.44 (<0.001) | 1.47 (0.0005) | 1.40 (0.003) | 1.52 (0.001) |
| Caregivers’ behavior factor score^d^ | 0.198 (0.148) | 0.173 (0.216) | 0.186 (0.169) | 0.224 (0.129) | 0.254 (0.049) |
| Caregivers’ attitude toward eggs^d^ | 0.186 (0.279) | 0.189 (0.293) | 0.197 (0.296) | 0.244 (0.179) | 0.293 (0.113) |
| Caregivers’ perceived benefits of eggs^d^ | 0.017 (0.885) | -0.002 (0.984) | -0.029 (0.817) | 0.060 (0.623) | 0.075 (0.559) |
| Social norms^d^ | 0.044 (0.659) | -0.019 (0.829) | -0.016 (0.870) | 0.016 (0.841) | 0.054 (0.376) |
| Caregivers’ self-efficacy^d^ | 0.325 (0.011) | 0.306 (0.011) | 0.339 (0.003) | 0.313 (0.038) | 0.297 (0.009) |
| Caregivers’ intent to feed eggs^d^ | 0.155 (0.027) | 0.161 (0.062) | 0.189 (0.014) | 0.174 (0.114) | 0.192 (0.022) |

Note: Analysis of covariance method and complex survey procedures were used to account for clustering at the local government area (LGA) level, adjusted for reported change in household consumption of foods due to the COVID-19 pandemic, and in all analyses the non-exposed are the participants from Kano state (n = 1,485).

^a^ Findings of the main analysis using the analysis of covariance method.

^b^ BTL activities include point-of-sale material deployment, neighborhood storm, market storm, school activation, primary health care, pantry raid and compound show, and World Egg Day and mobile egg truck.

^c^ Exposed sample includes participants who live within 2 km of BTL activity.

^d^ Effect is presented using regression coefficient and *p*-value.

^e^ Effect is presented using odds ratio and *p*-value.

Supplemental Table 5: Social desirability and outcome indicators by intervention group at end-line

| Outcome | Intervention group | Social desirability | | | | | |
| --- | --- | --- | --- | --- | --- | --- | --- |
|  |  | **Very low**  **(score = 0) (n = 231)**  **Mean ± SD or n (%)** | **Low**  **(score = 1) (n = 700)**  **Mean ± SD or n (%)** | **Medium (score = 2) (n = 876)**  **Mean ± SD or n (%)** | **High**  **(score = 3) (n = 897)**  **Mean ± SD or n (%)** | **Very high (score = 4–5) (n = 146)**  **Mean ± SD or n (%)** |  |
| Number of times eggs were consumed in the previous 7 days | Intervention | 1.18±1.66 | 1.41±1.89 | 1.33±1.74 | 0.98±1.32 | 0.66±1.26 |  |
|  | Comparison | 1.50±1.76 | 1.32±1.63 | 1.06±1.52 | 0.95±1.49 | 1.32±1.65 |  |
| Children consuming at least one egg in the previous 7 days | Intervention | 55(47.8) | 188 (50.7) | 220(50.9) | 169(45.2) | 17(25.4) |  |
|  | Comparison | 67(57.8) | 184 (56.1) | 201(45.5) | 221 (42.3) | 39(49.4) |  |
| Children consuming at least two eggs in the previous 7 days | Intervention | 36(31.3) | 145(39.1) | 158(36.6) | 109 (29.1) | 14(20.9) |  |
|  | Comparison | 50(43.1) | 117 (35.7) | 131(29.6) | 131 (25.1) | 29(36.7) |  |
| Eggs purchased in the previous month | Intervention | 77(66.9) | 242(65.1) | 281(64.8) | 236(63.1) | 35(52.2) |  |
|  | Comparison | 91(78.4) | 235 (71.6) | 289(65.4) | 304 (58.1) | 51(64.6) |  |
| Eggs acquired in the previous 7 days | Intervention | 53(46.1) | 174(46.8) | 227(52.3) | 158(42.3) | 32(47.8) |  |
|  | Comparison | 53(45.7) | 166 (50.6) | 191 (43.2) | 182(34.8) | 30(37.9) |  |
| Attitude score | Intervention | 0.70±0.81 | 0.61 ±0.82 | 0.55 ±0.78 | 0.53 ±0.81 | 0.76±0.72 |  |
|  | Comparison | 0.39±0.89 | 0.47 ±0.84 | 0.38±0.84 | 0.38 ±1.03 | 0.81 ±0.90 |  |
| Perceived benefit score | Intervention | 0.53±0.91 | 0.35 ±0.76 | 0.26 ±0.89 | 0.30 ±0.88 | 0.12±0.69 |  |
|  | Comparison | 0.19±0.92 | 0.23 ±0.76 | 0.30 ±0.80 | 0.35 ±0.80 | 0.54 ±0.80 |  |
| Norm score | Intervention | 0.42±0.73 | 0.31 ±0.73 | 0.31 ±0.89 | 0.24 ±0.89 | 0.13±0.70 |  |
|  | Comparison | 0.26±0.79 | 0.27 ±0.78 | 0.28±0.90 | 0.23 ±0.86 | 0.42 ±0.91 |  |
| Intent to feed eggs | Intervention | 3.99±0.85 | 3.97 ±0.82 | 4.07 ±0.84 | 3.98 ±0.96 | 3.91±0.75 |  |
|  | Comparison | 4.11±0.69 | 3.96 ±0.85 | 3.86 ±1.01 | 3.84 ±0.89 | 3.65 ±0.89 |  |
| Self-efficacy score | Intervention | 0.34±0.91 | 0.17 ±1.02 | 0.28 ±1.11 | 0.14 ±1.17 | 0.27±1.06 |  |
|  | Comparison | 0.24±0.96 | -0.07 ±1.12 | -0.20±1.15 | -0.13 ±1.08 | 0.32 ±1.29 |  |
| Behavior factor score | Intervention | 0.60±0.84 | 0.44 ±0.81 | 0.46 ±0.93 | 0.38 ±0.90 | 0.37±0.74 |  |
|  | Comparison | 0.38±0.92 | 0.29 ±0.90 | 0.22 ±0.93 | 0.24 ±0.93 | 0.53 ±0.88 |  |
